# Drop Behavior on Heterogeneous Ratchet-Structured Substrates Harmonically Vibrated in Lateral Direction

**DOI:** 10.1021/acs.langmuir.4c01563

**Published:** 2024-06-20

**Authors:** Rodica Borcia, Ion Dan Borcia, Michael Bestehorn

**Affiliations:** Institut für Physik, Brandenburgische Technische Universität, Erich-Weinert-Strasse 1, 03046 Cottbus, Germany

## Abstract

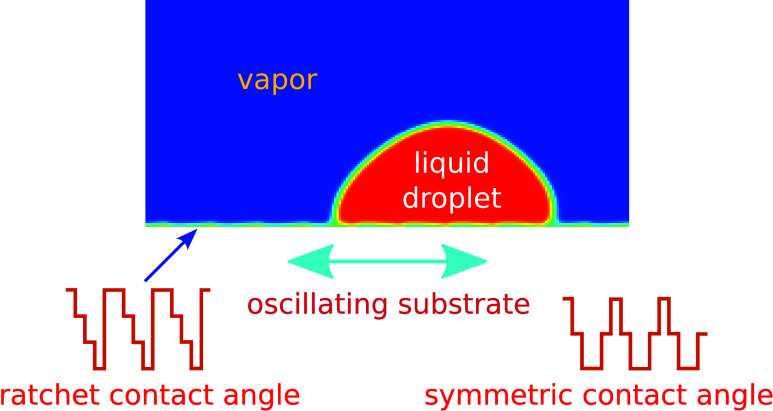

We analyze numerically a new ratchet system: a liquid drop is sitting on a heterogeneous ratchet-structured solid plate. The coated plate is subject to a lateral harmonic oscillation. The systematic investigation performed in the frame of a phase field model shows the possibility of realizing a long-distance net-driven motion for isolated domains of the forcing parameters. The studied problem might be of considerable interest for controlled motion in micro- and nanofluidics.

## 1 Introduction

Dynamics of sessile droplets on vibrated plates or on textured substrates is a ubiquitous phenomenon in our everyday experience, often leading to fascinating beautiful drop shapes.^1−3^ Wide applications of these problems are illustrated in literature, for example, in ink-jet printing, self-cleaning surfaces, microelectronics, microfluidic devices, or biomedical engineering (see ref (2) and references therein). Forcing vibrations can be experimentally realized by a camshaft driven by an electric motor^4^ or by electromagnetic vibration exciters (shakers).^5,6^ Textured surfaces can be realized chemically^7−11^ or opto-mechanically by femtosecond laser pulses.^12−14^

Drop-controlled motion is a valuable task in microfluidics. A possibility to obtain the manipulation of small liquid portions is to create a ratchet system. That means that the symmetry in the system has to be broken. To realize a droplet displacement along a solid substrate under inertial effects one can excite, for example, the solid plate by lateral and vertical simultaneous harmonic oscillation adjusted to the same frequency.^15,16^ The drop is driven by the substrate in one direction during one-half of a period (first half-cycle) and in the opposite direction during the other half (second half-cycle). Because the acceleration acting perpendicular to the substrate is pointing once up and then down during the two half-periods, the displacement to the left and to the right are not the same. This leads to a directional and long-distance net-driven motion. In ref (16), a parametric study has been performed for different excitation parameters and different wettabilities of the homogeneous solid plate. The drop shows a maximal response (displacement) for an excitation frequency related to the eigenfrequency of its elongated mode (which depends on the geometry and properties of the liquid droplet). The traveled distance depends on the wettability properties at the substrate as well: the resonant frequency increases with increasing surface hydrophobicity and saturates to a value close to the resonance frequency of the elongated mode. In ref (17), a mean mass flow emerges if the lateral excitation of the liquid film is asymmetric under time reversal, e.g., taking a ratchet shape excitation instead of a harmonic one. Here, drops formed due to Faraday instability of the flat film traveling in a distinguished direction have been observed.

In the present paper, we investigate a new ratchet mechanism: a liquid droplet sitting on a heterogeneous ratchet-structured substrate subject to a harmonic lateral vibration. The paper is organized as follows: We first introduce our mathematical and numerical model (Section 2). We then use computer simulation in two spatial dimensions to analyze this problem for different forcing parameters of the controlling bottom plate (Section 3). The validation test for liquid drop behavior on heterogeneous substrates has been also done. Different regimes of the net-driven motion have been identified and discussed. Finally, we present our conclusion remarks (Section 4).

## 2 Theoretical Framework

We study the drop behavior on a heterogeneous ratchet-structured substrate subject to a lateral oscillatory vibration employing a phase field model. The model was validated earlier for describing static and dynamic contact angles on homogeneous surfaces,^18^ dewetting phenomena, shaping of liquid films, controlled pattern formation,^19^ coalescence of drops,^20^ drop behavior on noisy surfaces,^12^ and drop motion under vibrations.^16^ Phase field models adopt a continuum thermodynamic description of multiphase systems: they introduce an order parameter (phase field variable) assumed to be nearly constant in every bulk region with a continuous and rapid variation from one phase to the other. In our model, the density ρ (scaled to the liquid density ρ_liq_) is the order parameter. ρ = 1 designates the liquid phase, and ρ ≈ 0 the vapor phase. The position of the interface is controlled by the gradients of ρ. For a two-phase system with diffuse interface, the Helmholtz free-energy functional contains two terms: the free-energy density for homogeneous phases *f*(ρ) and the gradient energy 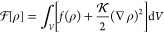
1where  denotes the gradient energy coefficient, a parameter related to the surface tension coefficient:  (see, e.g., refs (18,21)). Choosing a free-energy density of the form
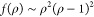
2one achieves the shape of a double well potential and one can model a system with two local minima corresponding to the two coexistent phases: liquid and vapor. As already shown in ref (18) or ref (21), minimizing the free-energy functional (1), one can derive the nonclassical phase field terms which have to be included in the Navier–Stokes equation for assuring the shear stress balance at the liquid–vapor interfaces. The corresponding Navier–Stokes equation incorporates therefore the Korteweg stress tensor^20−22^
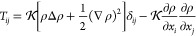
3where δ_*ij*_ is the Kronecker symbol. The numerical code is based on the Navier–Stokes equation with the Korteweg stress and the continuity equation
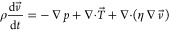
4
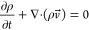
5with *p* = ρ∂*f*(ρ)/∂ρ – *f*(ρ) the dynamical pressure and η the dynamic viscosity. We analyze a two-dimensional (*xz*) dimensionless problem scaled according to ref (19). Similarly to ref (23), we apply an Euler method for the time integration. The spatial derivatives are discretized using a second-order central finite-difference scheme.^23^ Periodic boundary conditions in the horizontal direction are applied with no-slip condition for the velocity field *v⃗* at the top and bottom walls. The mesh is of 400 × 200 points, the distance between two mesh points is δ*x* = δ*z* = 2, and the integration time step is δ*t* = 0.1. The lengths are scaled to 10^–5^ m, and the time to 10^–5^ s. That means the computational domain is 8 mm × 4 mm. Integration arrays of more than 100 lattice points in each direction ensure an efficient numerical convergence and enough lattice points for a correct description of the diffuse interface.

**Figure 1:**
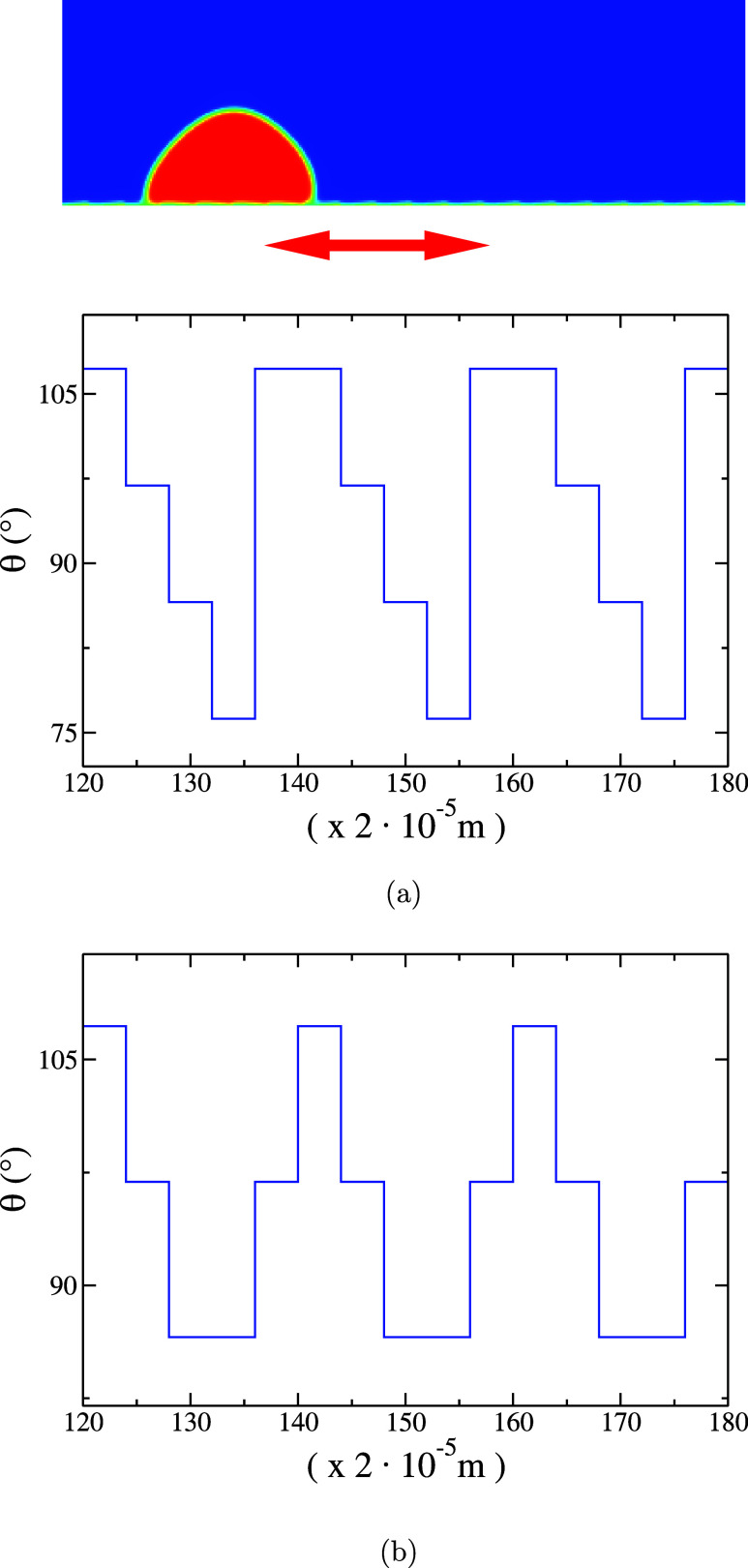
(a) Heterogeneous ratchet-structured substrate realized by varying θ(*x*) periodically. (b) Heterogeneous symmetrically-structured substrate realized by varying θ(*x*) periodically. Above we show only three periodic structures at the bottom boundary between the mesh points 120 and 180.

The density field is ρ = 10^–3^ at the top boundary and ρ = ρ_S_ at *z* = 0, simulating in this way at *z* = 0 a solid boundary with van der Waals long-range interactions at the liquid–solid interface.^21^ ρ_S_ is a free parameter between 0 and 1 which designates the fluid density at the substrate and describes the wettability properties at the bottom plate. 0 ≤ ρ_S_ ≤ 0.5 denotes hydrophobic surfaces (less precursor film at the solid substrate) and 0.5 < ρ_S_ ≤ 1 hydrophilic surfaces (much precursor film at the solid boundary). The static contact angle θ is related to the density ρ_S_ at the substrate through^21^
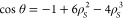
6The initial condition is a horizontal thin liquid film (with ρ = 1) of height *h*_0_ = 0.4 mm. The rest of the closed domain is filled with vapor (with ρ ≈ 0). At the initial moment, there is no motion in the system (*v⃗* = 0). The thin liquid film is unstable and breaks up into several droplets. The surface tension drives the coalescence and finally one gets only one single drop at rest sitting on the solid substrate. By changing ρ_S_, one obtains droplets of the same mass  with different contact angles at the bottom plate. The droplet radius in two spatial dimensions is calculated as . For the numerical results presented in this paper, we have circular drops of radius *R* = 0.8 mm, ρ_liq_ = 1000 kg/m^3^, η = 0.001 kg/ms, and σ = 0.05 N/m. A heterogeneous ratchet-structured substrate can be experimentally realized through chemical patterning, for example. In our simulation, it is enough to obtain this patterning by varying ρ_S_, resulting (by using formula 6) to θ(*x*) displayed in Figure 1(a). The substrate structured according to Figure 1(a) is subject to a harmonical oscillatory excitation in lateral direction *v*_plate_ = *A*ω sin ω*t*, with *A* being the excitation amplitude and ω the angular frequency (*A*ω is scaled to 0.1 m/s). We take the bottom boundary as frame of reference (i.e., we have a noninertial frame of reference), which imposes to include the pseudo-force, −ρd*v*_plate_/d*t* on the right-hand side of the momentum eq 3 in the *x* direction.

## 3 Numerical Results and Discussion

**Figure 2:**
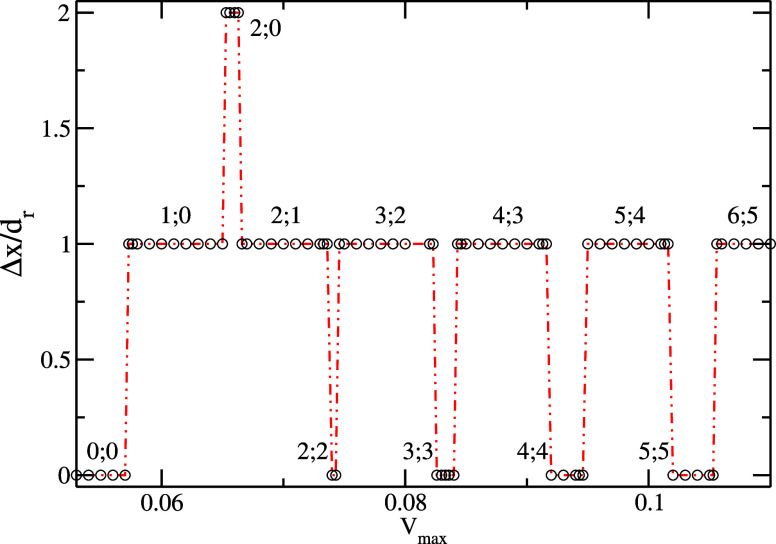
Droplet displacement (per excitation period) versus the amplitude *V*_max_ = *A*ω (ω = 2.6 ·10^–5^, *d*_r_ = 40). The scaled time is 10^–5^ s, the scaled length is 10^–5^ m, and the scaled velocity is 0.1 m/s.

The liquid droplet is sitting on a striped coated substrate with contact angles varying between θ = 76° and θ = 107° (see Figure 1) covering 4 striped structures. The spatial periodicity of the patterned substrate is *d*_r_ = 40. We analyze the location of the drop’s mass center
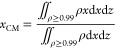


over 7 periods of the lateral forcing oscillations at the bottom boundary. For all of the simulations presented in this paper, the droplet is initially located in the same position on the substrate. The initial droplet is provided by the numerical simulation with ρ_S_ = 0.4 (θ = 107°) at the solid substrate. The droplet follows a swinging movement superimposed on a movement in the horizontal direction. The net horizontal displacement can be separated by using a stroboscopic method: we consider the drop position at the same phase of the forced oscillation. Figure 2 plots the traveled distance of the drop per period versus *V*_max_ = *A*ω for an angular frequency ω = 2.6 ·10^–5^. For small forcing amplitudes *A*ω ≤ 0.057, little swayings caused by inertial effects occur, but the droplet remains pinned at the substrate. Therefore, the liquid drop does not move along the substrate [regime (0;0) in Figure 2]. As one can see in Figure 1(a), the shape of the curve θ(*x*) is asymmetric along one spatial period, suggesting a ratchet. That means there are different pinning forces at the substrate when the droplet is pulled to the right or to the left. For a velocity amplitude of 0.0573, the droplet is pulled to the next coated stripe to the right during the forcing at the substrate to the left (first half-cycle). But it remains further pinned at the substrate during the forcing in the opposite direction (second half-cycle), i.e., the drop does not advance to the left. This is the regime marked with (1;0) in Figure 2. Since the droplet motion relative to the substrate is uncompensated during one period of the forced vibrations, the droplet advances with one spatial periodicity *d*_r_ (one step) to the right relative to the solid plate per excitation period. The net-driven motion appears at each forcing oscillation at the substrate so that after several oscillations, one can achieve a long-distance controlled motion of the liquid drop. In this way, one can realize a ratchet-driven motion along the patterned substrate. For excitation amplitudes between 0.0653 ≤ *A*ω ≤ 0.0663, the droplet experiences a regime characterized by two steps to the right and no step to the left during one period. So, the regime (2;0) marked in Figure 2 allows the possibility of a 2 times faster droplet transport along the solid plate. By increasing the excitation amplitude, one reaches the regime when the inertial force during the forcing to the right becomes strong enough to detach and move also the droplet to the left. Therefore, we get the regime (2;1) illustrated in Figure 2, i.e., creating again a driven motion of one spatial periodicity per one forcing period. By continuing to increase the amplitude, one gets the situation when the droplet is able to move two stripes to the right and also two stripes to the left, i.e., “0”-driven motion during one excitation period [regime (2;2) in Figure 2]. How one can see in Figure 2, we can also get the regimes (3;2), (3;3), (4;3), (4,4), (5;4), (5;5), (6;5), etc. Therefore, one remarks that driven motion can occur only for isolated domains of velocity amplitudes (“bands of motion”).

**Figure 3:**
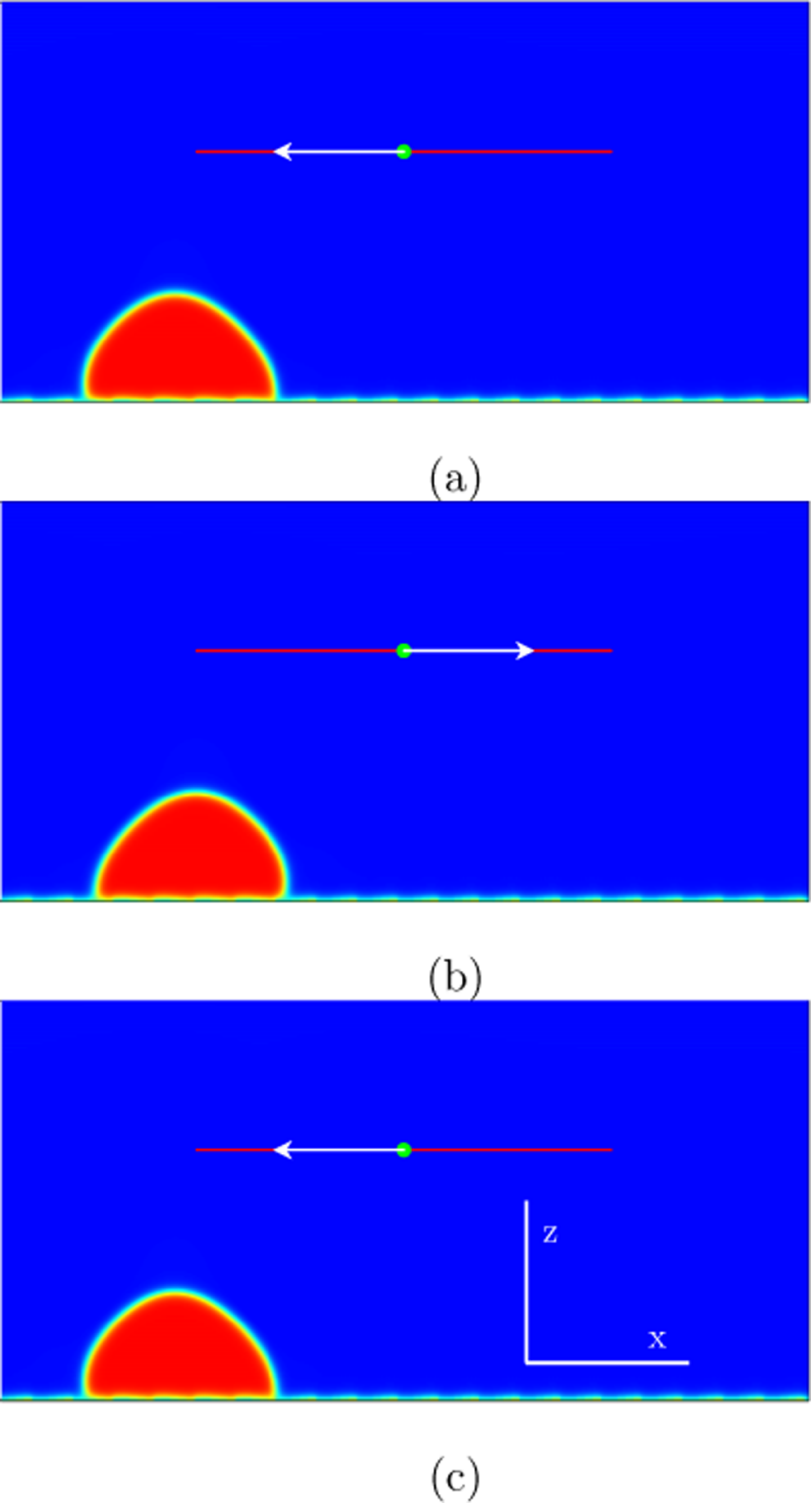
Numerical simulations in two spatial dimensions showing the behavior of the liquid drop during a period for the mode (0,0) marked in Figure 2: (a) *t* = 240,000; (b) *t* = 361,000; (c) *t* = 482,000. The patterning at the substrate is applied according to Figure 1 with contact angles varying between θ = 76° and θ = 107°. Hydrophobic surfaces mean less precursor film at the solid substrate and hydrophilic surfaces much precursor film at the solid boundary. The excitation amplitude of the forcing velocity is *A*ω = 0.05 and the excitation frequency ω = 2.6 ·10^–5^. Little drop “swaying” (“dancing”) under inertial effects can be observed, but during an excitation period, the drop does not leave its initial position.

**Figure 4:**
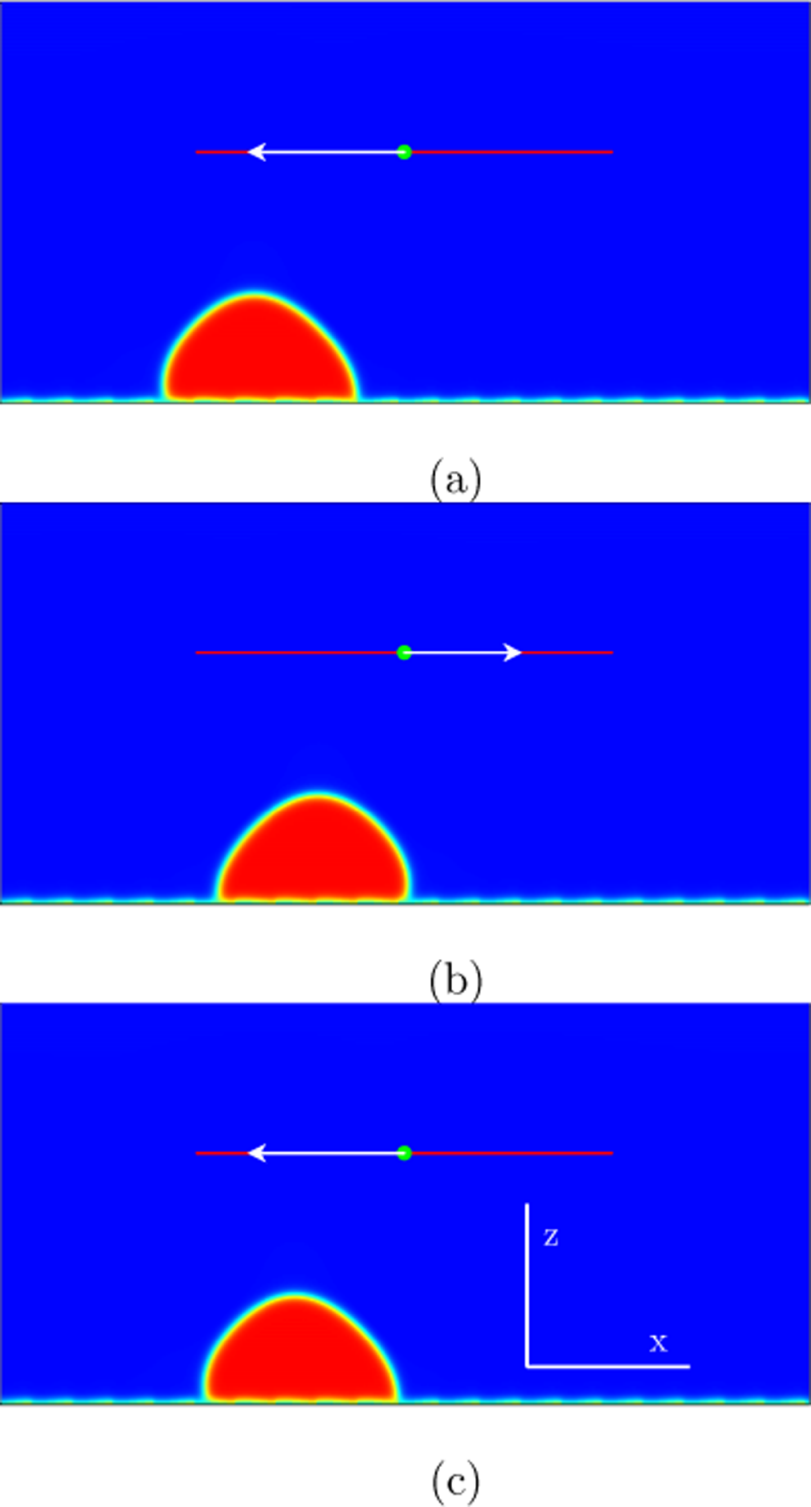
Numerical simulations in two spatial dimensions showing the behavior of the liquid drop during one period for the mode (1,0) marked in Figure 2: (a) *t* = 482,000; (b) *t* = 632,000; (c) *t* = 723,000. The excitation amplitude is *A*ω = 0.06 with ω = 2.6 ·10^–5^. When the solid plate is pulled to the left (first half-cycle), the drop advances with one spatial periodicity to the right, but does not leave its spot, when the solid plate is pulled to the right (second half-cycle). In this way, the drop advances one periodical structure (one step) to the right per each oscillation relative to the bottom plate.

**Figure 5:**
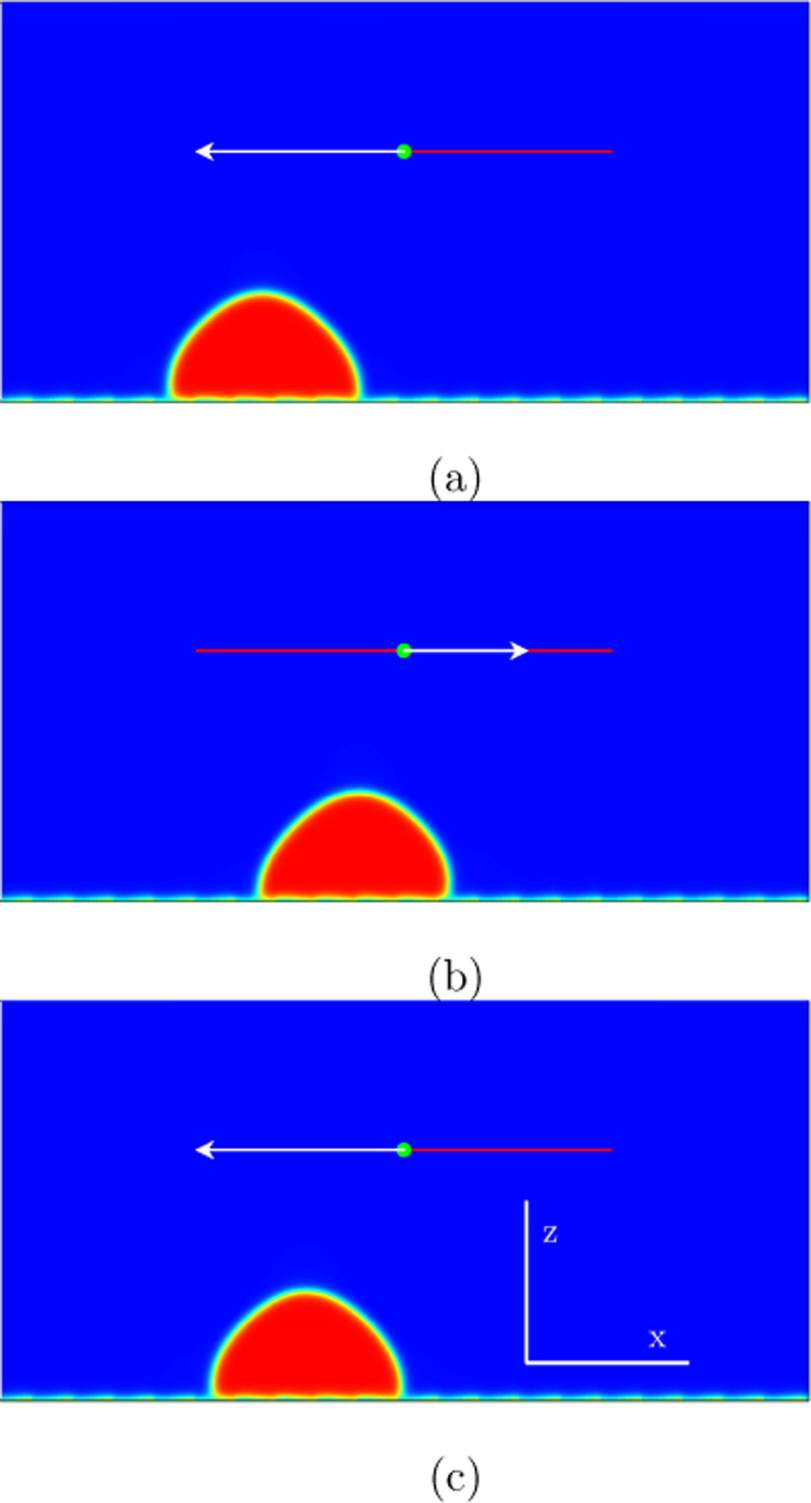
Numerical simulations in two spatial dimensions showing the behavior of the liquid drop during one period for the mode (3,2) marked in Figure 2: (a) *t* = 482,000; (b) *t* = 640,000; (c) *t* = 723,000. The excitation amplitude is *A*ω = 0.08 with ω = 2.6 ·10^–5^. The drop moves forth and back during a period: 3*d*_r_ (three steps) to the right in the first half-cycle and 2d_r_ (two steps) to the left in the second half-cycle. That means per one excitation period one spatial periodicity *d*_r_ (one step) to the right.

**Figure 6:**
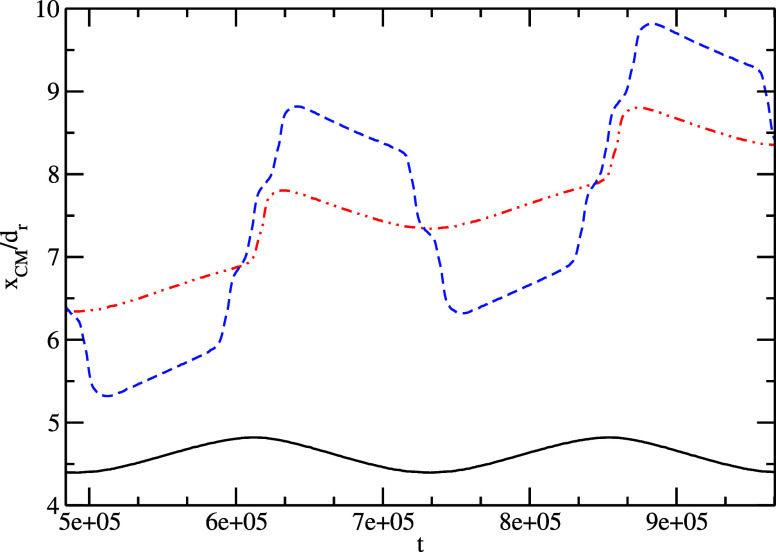
Horizontal position of the drop mass center for three different excitation amplitudes: *Aω* = 0.05 (black solid line), *A*ω = 0.06 (red dash–dotted line), and *A*ω = 0.08 (blue dashed line) for the snapshots depicted in Figures 3–5, respectively (ω = 2.6 ·10^–5^, *d*_r_ = 40). The scaled time is 10^–5^ s, the scaled length is 10^–5^ m, and the scaled velocity is 0.1 m/s. The plot has been done for two excitation periods.

We plot the density profiles for different regimes. Snapshots with drop behavior during one period are emphasized in Figures 3, 4 and 5 for, respectively, (0;0), (1;0), and (3;2) regimes (see also the corresponding movies available as additional supporting material). The arrows shown in Figures 3–5 follow the elongation of the lateral harmonical excitation motion at the bottom boundary. For the cases depicted in Figures 3–5, we represent in Figure 6 the time evolution of the drop mass center over two excitation periods at the solid boundary. For the mode (0;0), corresponding to the smallest excitation amplitude *A*ω = 0.05, the curve shows a periodic harmonic function. The oscillation amplitude is not high enough to move the drop over the pinning regions. The evolution of the mass center position is slightly delayed to the excitation acceleration. For the mode (1;0), the excitation amplitude *A*ω = 0.06 is high enough to move the drop over the right pinning region but not enough to move over the left one, where the pinning is stronger due to the higher gradient of the surface tension. The moment of detachment can be observed in Figure 6 at the moment *t* = 609,250 and again at *t* = 850,750, after one period. At those time moments, the drop starts to move faster and therefore the derivative of the curve has for a short time a much higher value. For the mode (3;2), the excitation amplitude *A*ω = 0.08 is high enough to overpass two left pinning regions, but it can move over three pinning regions to the right. This also results in the same mean displacement of one length unit during an oscillation period. On the plot, one can see five jumps for this case, three to the right and two to the left, with small regions in between, where the derivative of the curves decreases for a short time.

We notice that the frequencies in all of the simulations are several orders of magnitude lower than the eigenfrequency of the elongated mode. Consequently, the resonance effects are not involved in the drop behavior in this paper.

**Figure 7:**
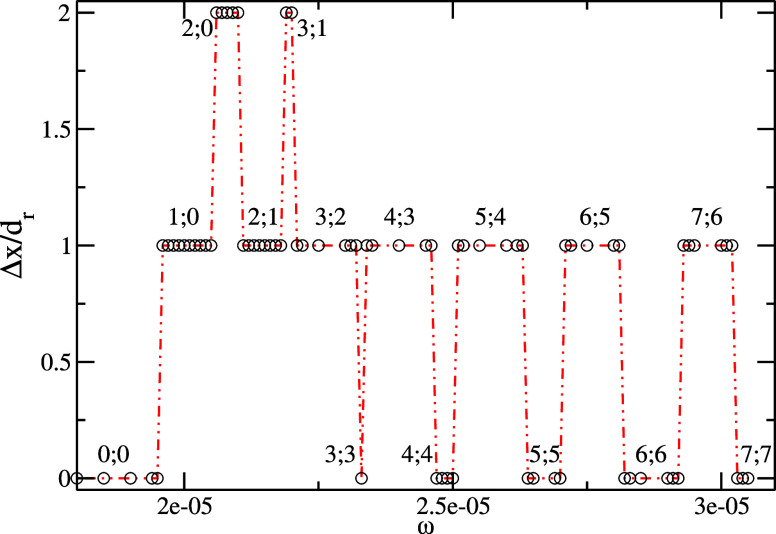
Droplet displacement (per excitation period) versus the excitation angular frequency ω for an excitation amplitude *V*_max_ = *A*ω = 0.1 (*d*_r_ = 40). The scaled time is 10^–5^ s, the scaled length is 10^–5^ m, and the scaled velocity is 0.1 m/s.

**Figure 8:**
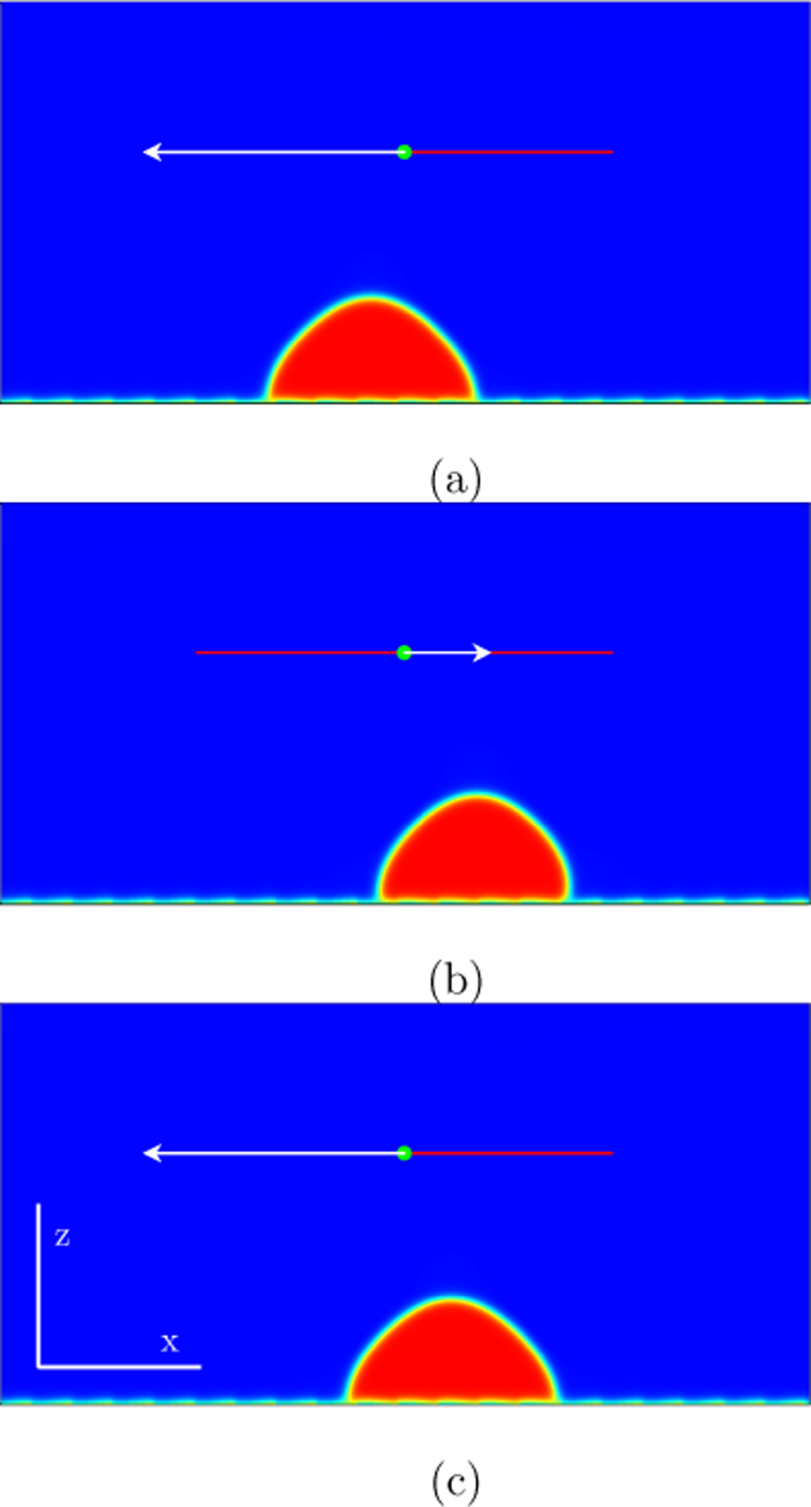
Numerical simulations in two spatial dimensions showing the behavior of the liquid drop during one period for the mode (3,1) marked in Figure 7: (a) *t* = 570,000; (b) *t* = 770,000; (c) *t* = 856,000. The amplitude of the forcing velocity at the substrate is *A*ω = 0.1 and the excitation frequency ω = 2.2 ·10^–^^5^. The drop moves three spatial periodicities 3d_r_ to the right in the first half-cycle and one spatial periodicity *d*_r_ to the left in the second half-cycle. That means per one excitation period 2d_r_ to the right relative to the patterned substrate.

Figure 7 plots the motion diagram for an excitation amplitude *A*ω = 0.1 and the ratchet–structured patterning at the substrate according to Figure 1. The dependency of the drop traveled distance versus the excitation frequency for the ratchet-structured substrate reveals again the motion in “bands”, most 0 or *d*_r_. As in Figure 1, we identify two fast motion regimes: (2:0) and (3;1). In the regime (3:1), the droplet experiences three steps to the right and one to the left, i.e., 2*d*_r_ to the right relative to the solid plate during one forced oscillation. A series of snapshots for this regime (3;1) are illustrated in Figure 8 (the corresponding video is also available in the Supporting information).

As validation test for the heterogeneous substrates, one considers the symmetrically-structured substrate, patterned according to Figure 1(b) [with θ (*x*) having a symmetrical shape per one spatial period with contact angles varying between θ = 86° and θ = 107°]. As expected, we find “0”-driven motion for different angular excitation frequencies.

## 4 Conclusions

We have systematically studied a new ratchet system: the droplet motion on a heterogeneous ratchet-structured substrate subject to a lateral harmonic oscillation. In this aim, a phase field tool has been employed. Our predictions show long-distance net-driven motions for isolated domains of the harmonical forcing parameters at the bottom boundary. The studied problem has huge interest for applications in microfluidics and microgravity, where the role of the gravity effects are negligible—while the role of the inertial effects induced by vibrations becomes significant—and paves the way to future studies in this direction.

## Supporting Information Available

Temporal behavior of liquid droplets over several excitation periods at the ratchet-structured solid substrate for the regime presented in Figure 3 (AVI)
Temporal behavior of liquid droplets over several excitation periods at the ratchet-structured solid substrate for the regime presented in Figure 4 (AVI)
Temporal behavior of liquid droplets over several excitation periods at the ratchet-structured solid substrate for the regime presented in Figure 5 (AVI)
Temporal behavior of liquid droplets over several excitation periods at the ratchet-structured solid substrate for the regime presented in Figure 8 (AVI)

## Supplementary Material

la4c01563_si_001.avi

la4c01563_si_002.avi

la4c01563_si_003.avi

la4c01563_si_004.avi
